# Facile one-pot synthesis of flower-like AgCl microstructures and enhancing of visible light photocatalysis

**DOI:** 10.1186/1556-276X-8-442

**Published:** 2013-10-24

**Authors:** Meicheng Li, Hang Yu, Rui Huang, Fan Bai, Mwenya Trevor, Dandan Song, Bing Jiang, Yingfeng Li

**Affiliations:** 1 State Key Laboratory of Alternate Electrical Power System with Renewable Energy Sources, School of Renewable Energy, North China Electric Power University, Beijing 102206, China; 2 Suzhou Institute, North China Electric Power University, Beijing 102206, China; 3 School of Materials Science and Engineering, Harbin Institute of Technology, Harbin 150001, China

**Keywords:** AgCl, Flower-like microstructures, Visible light, Photocatalytic

## Abstract

Flower-like AgCl microstructures with enhanced visible light-driven photocatalysis are synthesized by a facile one-pot hydrothermal process for the first time. The evolution process of AgCl from dendritic structures to flower-like octagonal microstructures is investigated quantitatively. Furthermore, the flower-like AgCl microstructures exhibit enhanced ability of visible light-assisted photocatalytic degradation of methyl orange. The enhanced photocatalytic activity of the flower-like AgCl microstructure is attributed to its three-dimensional hierarchical structure exposing with [100] facets. This work provides a fresh view into the insight of electrochemical process and the application area of visible light photocatalysts.

## Background

The semiconductor-mediated photocatalytic decomposition of organic pollutions in the environment has attracted much attention [1] because of the abundant available solar resources and the minimum requirements of carbon footprint generated. Among the various semiconductor photocatalysts, TiO_2_ is the most extensively employed photocatalyst, owing to its high photocatalytic activity, good chemical stability, non-toxicity, and low cost. However, TiO_2_ absorbs only ultraviolet light, which accounts for only 4% of the total sunlight. Since about 48% of sunlight is visible light, it is strongly important to develop the photocatalysts which are active and effective under visible light.

As a well-known material used for photographic film, AgCl has shown its valuable applications as visible light photocatalysts [2-8]. AgCl is a stable photosensitive semiconductor material with a direct band gap of 5.15 eV and an indirect band gap of 3.25 eV. Although the intrinsic light response of AgCl is located in the ultraviolet region as well, once AgCl absorbs a photon, an electron-hole pair will be generated and subsequently, the photogenerated electron combines with an Ag^+^ ion to form an Ag atom [7]. Finally, a lot of silver atoms are formed on the surface of the AgCl, which could extend the light response of AgCl into the visible light region [1,6,7].

Besides, the morphology of AgCl has significant influence on its photocatalytic activity, so it is important to develop facile methods to synthesize size- and shape-controlled AgCl materials. Recently, the facile hydrothermal method is employed to synthesize variable micro-/nano-AgCl structures, including AgCl nanocubes [6], cube-like Ag@AgCl [7], and even near-spherical AgCl crystal by an ionic liquid-assisted hydrothermal method [8]. However, for AgCl microcrystals, this narrow morphology variation (simply varied from near-spherical to cubical [2-7]) inspired that more particular attention is deserved to pay on the novel AgCl morphologies, including the synthesis methods and their generation mechanisms, even the possible morphology evolution processes.

Herein, the novel flower-like AgCl microstructures similar to PbS crystals [9] are synthesized by a facile hydrothermal process without any catalysts or templates. Also, a series of AgCl morphology evolution processes are observed. Flower-like structures are recrystallized after the dendritic crystals are fragmentized, assembled, and dissolved. The detailed mechanism of these evolution processes has been further discussed systemically. Furthermore, flower-like AgCl microstructures exhibited enhanced photocatalytic degradation of methyl orange under visible light.

## Methods

The AgCl dendritic and flower-like structure are synthesized via hydrothermal method by reacting silver nitrate (AgNO_3_, 99.8%) with ethylene glycol (EG, 99%) in the presence of poly(vinyl pyrrolidone) (PVP-K30, MW = 30,000). In a typical synthesis, all the solutions are under constant stirring. Firstly, a 10-ml EG solution with 0.2 g of PVP was prepared. Then using droppers, another 7 ml of EG which contained 10 mM of AgNO_3_ is added. Afterwards, 3 ml of undiluted hydrochloric acid (HCl, 36% ~ 38%) is added into this mixture. The mixed AgNO_3_/ PVP/HCl/EG solution is further stirred for several minutes until it becomes uniform. This solution is then transferred into a 25-ml Teflon-lined autoclave tube and dried in the drying tunnel at 160°C for different times. The final products are collected by centrifugation (6,000 rpm, 10 min) and washed several times with deionized water.

The electron scanning microscopy (SEM) measurements are obtained on FEI Quanta 200F microscope (FEI Company, Hillsboro, OR, USA). The X-ray powder diffraction (XRD) patterns of samples are examined by Bruker D8 focus X-ray powder diffractometer (Bruker Corporation, Billerica, MA, USA) with Cu Kα radiation at λ = 1.5406 Å. Photocatalytic degradation of organic dye methyl orange (MO) is conducted under visible light at room temperature with a prepared solution of 100 mg/L AgCl powder and 20 mg/L MO dye in a 100-ml beaker. The concentration of MO in the solution is tested with a UV-vis spectrophotometer (UNICO UV-2450; UNICO, Dayton, NJ, USA).

## Results and discussion

**Figure 1:**
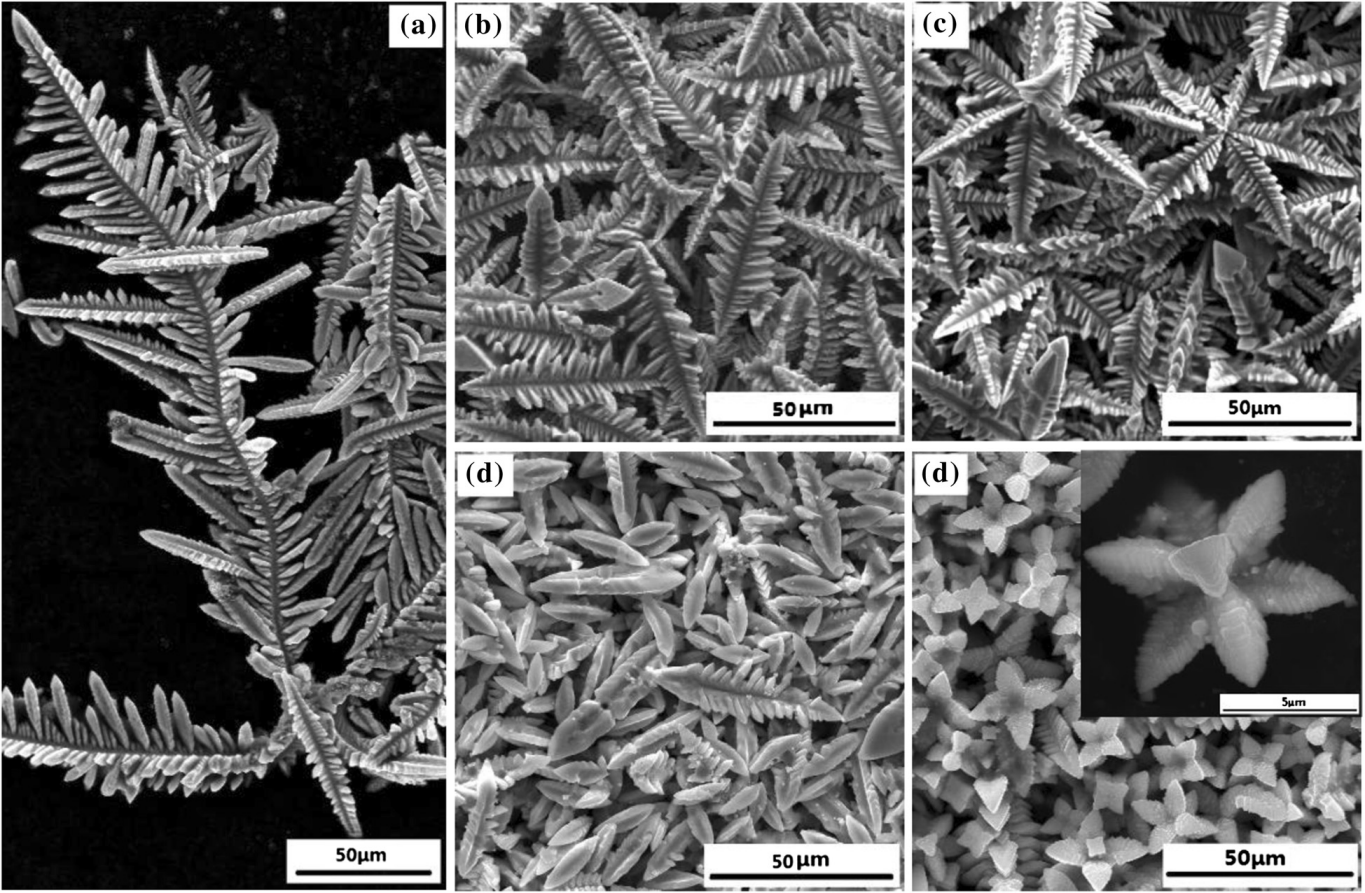
**SEM images of AgCl microstructures prepared by one-pot hydrothermal process at different reaction times. (a)** The big AgCl crystal dendrites formed after 3 h of reaction. **(b)** The big dendrites fragmentized into smaller dendrites after 6 h of reaction. **(c)** The eight smaller dendrites assembled on each corner of a cube to develop symmetric octagonal dendrites after 7 h of reaction. **(d)** The sub-dendrites of the octagonal dendrites dissolved to smaller and smoother sub-dendrites after 9 h of reaction. **(e)** The final products were the symmetric flower-like AgCl microstructure crystals after 11 h of reaction; the insert is the amplified image.

Herein, a novel flower-like AgCl microstructure is synthesized by a facile hydrothermal process without any catalysts or templates, as shown in the SEM image in Figure 1e and the insert with amplified view. Confirmed by the XRD patterns, the as-prepared sample exhibits a cubic AgCl structure (JCPDS no. 31-1238) with lattice constant *a* = 5.5491.

During the synthesis process, AgCl crystals are mainly formed through reaction (1). It is found that the concentration of Cl^-^ plays a vital role in the final shape of AgCl, because both cubic and concave cubic AgCl crystals can be obtained by varying the concentration of Cl^-^[2]. So, we added considered HCl in the synthesis process. Meanwhile, as AgCl is not stable under the circumstance with the excess concentration of Cl^-^, a reversible reaction (2) could happen in this circumstance to generate coordination compound [AgCl_2_]^-^:

(1) $${\mathrm{AgNO}}_{3}+\mathrm{HCl}=\mathrm{AgCl}↓+{\mathrm{HNO}}_{3}$$

(2) $$\mathrm{AgCl}+{\mathrm{Cl}}^{-}={\left[{\mathrm{AgCl}}_{2}\right]}^{-}$$

**Figure 2:**
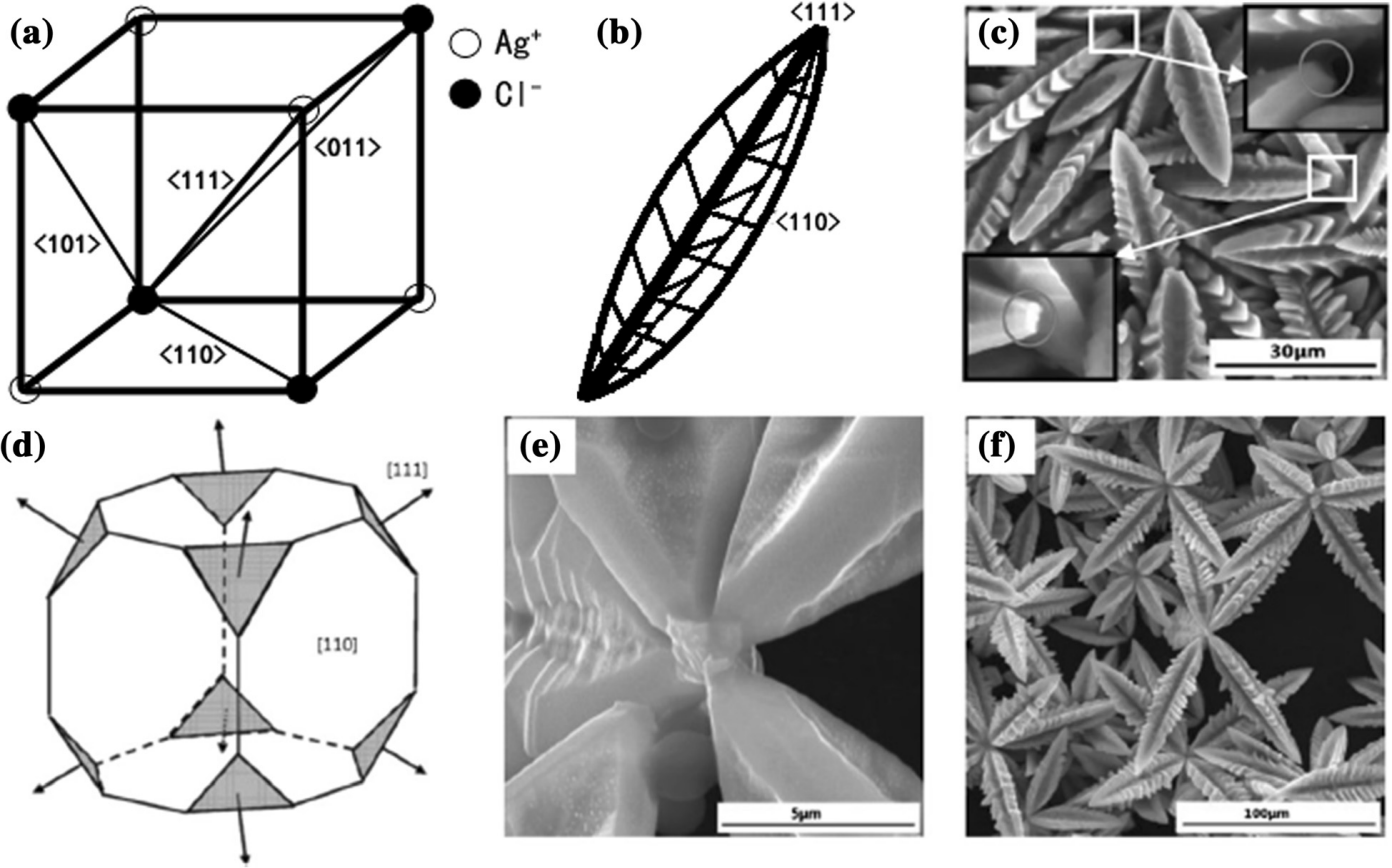
**Morphologies of the products that evolved with the reaction time. (a)** A crystal cell describing the main direction and three sub-directions. **(b)** Schematic diagram of the dendritic AgCl showing the dendrite's trunk grow along <111> direction. **(c)** SEM image of AgCl sub-dendrites; the insets are the amplified pictures of the two squares, and the roots of the sub-dendrites are plane. **(d)** Schematic diagram of the cube at the assembling center with eight plane [111] faces. **(e)** High-resolution SEM images of the octagonal assembled site. **(f)** SEM image of the assembled octagonal dendritic AgCl crystal structures.

Based on the equations, AgCl dendritic crystals and flower-like structures are synthesized. Meanwhile, we found that the morphologies of the products are gradually evolved with the reaction time, as shown in Figure 2a,b,c,d,e. A trend of regular morphology evolution from shiftable dendritic combinations to flower-like crystals is obvious as well.

At the first stage, the dendritic AgCl crystal structures are composed when the reagent concentration is very high. As we know, according to the crystal growth theory, under a certain concentration, the fastest growth face would fade away earliest while the crystal was growing. Besides, AgCl crystals have preferential overgrowth along <111> and then <110> direction based on the previous work [2]. Hence for AgCl crystal, when the reactants’ concentration are below a certain value, the [111] face would finally disappear and leave [110] face presented, thus forming cubic-faceted crystals; however, if the concentration were above the critical value, crystals would grow along [111] face, therefore forming dendritic crystals. This is the reason that dendritic structures are more likely to be generated during the early period while cubic structures are preferred in the subsequent period. As described in Figure 1a, we obtained dendritic crystals with the reaction time of 3 h.

Meanwhile, in Figure 1a, it can be seen that the initial dendrites are so large that their lengths expand to several hundred micrometers. However, the small branches would separate from the trunk, as many sub-branch arms showed in Figure 1b. These small branches own the same size and morphology with the sub-branch in Figure 1a. We can also observe from Figure 1a that shorter sub-dendrites are more robust and ordered than longer sub-dendrites when attached alongside the main truck. So longer side branches are more easily to fragmentize. Similar branch-breaking phenomenon has been observed in Ag dendrites [10]. Actually, several reasons can contribute to these results. First, not only large-size dendrites create greater stress in the connections between sub-branches and the trunk, but also a larger branch distance decreases the interactions among each sub-branch. Additionally, a high growth speed is inclined to compound-multiply twinned dendrites which are more active and impressionable to be modified. As a whole, all of these are immersed in heat convection surroundings that create a flowing condition for branch fragment.

After the first stage, the crystal growth model of AgCl changes due to the reduction of reagent concentration to a certain value. Then cubic-faceted crystals are easier to synthesize than dendritic crystals. The new growing cubic and original dendritic crystals would integrate into assembled dendrites in Figure 1c. In the process, we find that all the dendrites are well organized with three faces of sub-branches, owing to the specific AgCl crystal structure as shown in Figure 2a,b. From the insert images in Figure 2c, we can see that the sub-branch dendritic root is plane, the surface is the [111] face. Thus, during the formation of a cubic structure, there is a period that the eight [111] faces are exposed outside, just as shown in Figure 2c and the scheme image in Figure 2d. Because of the co-existing of two similar crystallographic orientation faces, the two faces would attach together thermodynamically in order to eliminate the pairs of high-energy surface. This is exactly the so-called oriented attachment mechanism [11]. After the assembling of the sub-branch dendrites, ions in the solution continue to aggregate around the assembled sites to form robust AgCl-assembled dendrites (Figure 2e,f). As a result, assembled dendrites with eight branches are created, which we call octagonal dendrites, as shown in Figure 1c.

As we know, nucleating and dissolving simultaneously take place throughout the whole reaction process, and their rates changed constantly. The reagent concentration decreases as the reaction time prolonged, so the rate of nucleating becomes slow and reaches equilibrium with the dissolving rate. Basically, no extra amount of AgCl crystal is generated under this circumstance.

However, due to the extremely high concentration of HCl, a third round of instability referring to Equation 2 is underlying. So the octagonal dendrite dissolves into eight dendrites and their surface becomes smoother. Therefore, smaller smooth dendrites (20 to 30 μm compared with 50 to 60 μm before) are generated as showed in Figure 1d.

**Figure 3:**
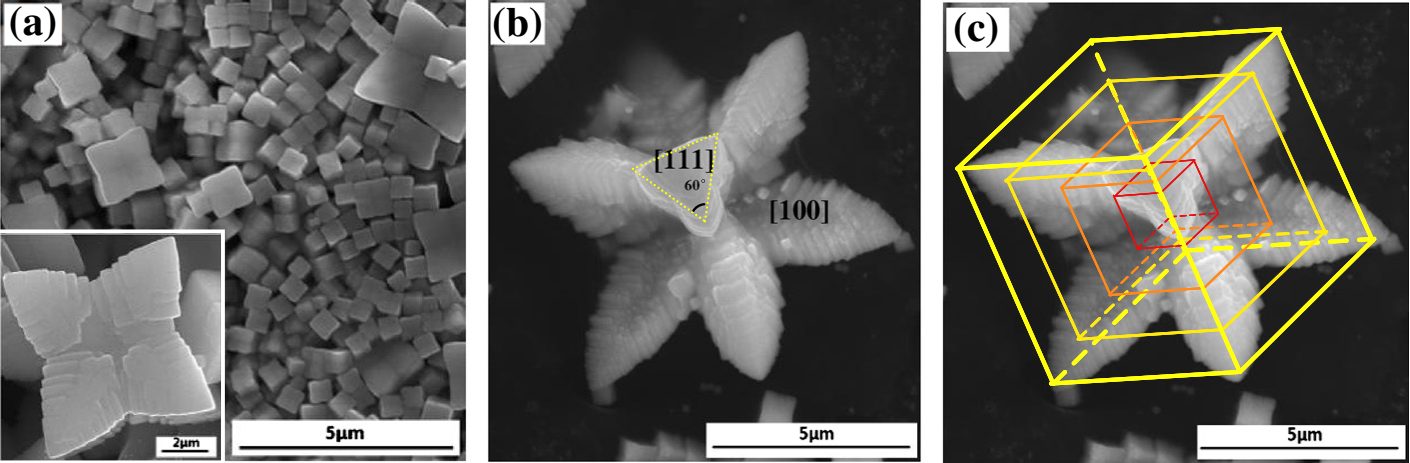
**SEM images of AgCl microstructures by one-pot hydrothermal process at different reaction times. (a)** After 10 h of reaction, abundant nucleated AgCl cubic seeds appear, which began to show preferential overgrowth along <111> direction; insert is a growing 'flower’ preferential growth from a cubic-faceted crystals seed. **(b,c)** The same image with different schematic labels, which is the cube in **(a)** grows to symmetric flower-like octagonal crystals after 11 h of reaction.

From the above analysis, we know that the processes of the dissolving and the nucleating ran into another round, and at this time, the appearance of new ions generated by dissolving could contribute to the new round of nucleating. However, the concentration of the new product ion is not high enough to generate dendritic crystals; cubic seeds form instead as shown in Figure 3a. At this time, there is no fragmentized dendrite with plane roots co-existing, so no assembling process occurs. The new product ions constantly stick to the new forming cubes. As shown in the insert of Figure 3a, a large number of new generating cubes receive ions, and then preferentially overgrow along the direction of <111> and secondary along <110> orientation. That is actually the same orientation choice as the other study has shown [2]. Therefore, the flower-like octagonal crystals formed as shown in Figure 3b, which dimension is smaller than the previous octagonal dendrites. Meanwhile, there are a large number of uniformly distributed step structures as shown in the insert of Figure 3a. This kind of three-dimensional hierarchical structure has another characteristic: all the faces of the step structure expose [001] facets. The hierarchical structure is expected to have high superficial area. As a consequence, a novel structure, which is flower-like with eight petals, is generated as shown in Figure 1e and Figure 3b.

**Figure 4:**
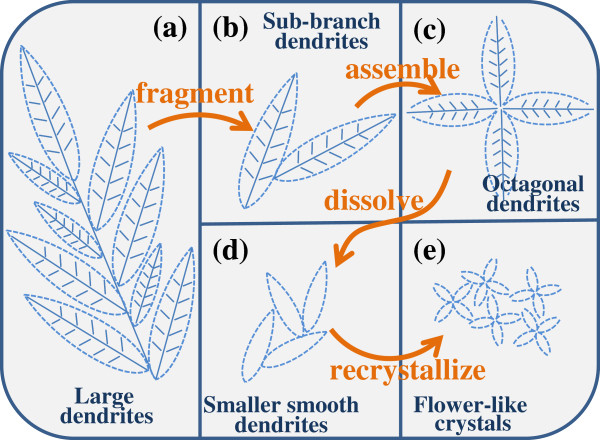
Schematic illustration of the evolution process of AgCl dendritic structure to flower-like octagonal microstructures.

Above all, the whole morphology evolution process of AgCl crystals is elucidated in detail. The schematic illustration of the evolution process of AgCl dendritic structure to flower-like octagonal microstructures is shown totally in Figure 4. Crystal growth dynamics, dissolving and nucleating processes, etc. alternate among the synthesis process, and together they provide a novel evolution mechanism. To an extent, this morphology evolution process enriches the research field of AgCl and other related crystals.

Apart from the detailed analyzing of the growth mechanism of the flower-like AgCl microstructures, the photocatalytic performance of the AgCl microstructures also has been evaluated with the decomposition of MO, under the illumination of the visible light. In fact, the decomposition of organic contaminant happened because the light-induced oxidative holes are generated around the MO molecules when the AgCl microstructures are exposed to sunlight.

**Figure 5:**
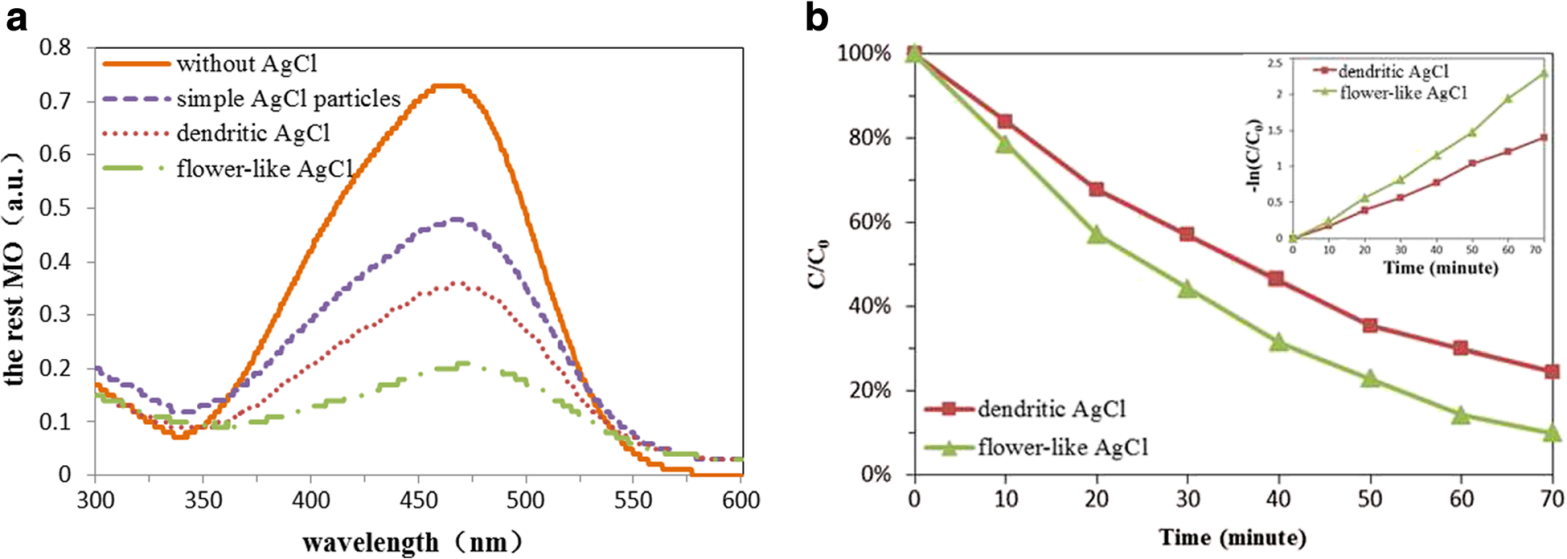
**UV-visible spectra of MO and comparison of its concentration. (a)** The UV-visible spectrum of MO dye after the degradation time of 1 h in solution over simple AgCl particles, dendritic AgCl, flower-like AgCl, and without AgCl. **(b)** The variation of MO concentration by photoelectrocatalytic reaction with dendritic and flower-like AgCl octagonal microstructures, i.e., the comparison of the degradation rates.

We measure several crystals’ photocatalytic properties under the same conditions. Figure 5(a) shows UV-visible spectrum of MO dye after the degradation time of 1h in solution over simple AgCl particles, dendritic AgCl, flower-like AgCl and without AgCl. It can be seen that the peak intensity decreases rapidly at the wavelength of 464nm, which correspond to the functional groups of azo [12]. We found that 80 % of MO molecules can be degraded by the flower-like AgCl. From the comparison curves, it can clearly see that both dendritic AgCl and flower-like AgCl exhibit much stronger photocatalytic activity in the visible light than that of AgCl particles. Also the photocatalytic efficiency of flower-like AgCl is the highest in these four types of samples.

Figure 5b shows the linear relationship of lnC_0_/C vs. time. We can see that the photocatalytic degradation of MO follows pseudo-first-order kinetics, lnC_0_/C = *kt*, where C_0_/C is the normalized MO concentration, *t* is the reaction time, and *k* is the pseudo-first-rate constant. The apparent photochemical degradation rate constant for the flower-like AgCl microstructure is 3.38 × 10^-2^ min^-1^, which is almost two times that for the dendritic AgCl, 1.87 × 10^-2^ min^-1^. This further confirms that flower-like AgCl microstructures exhibit higher photocatalytic efficiency. Overall, the flower-like AgCl microstructures exhibit excellent photocatalytic activity under visible light irradiation.

The enhanced photocatalytic activity of the flower-like AgCl microstructure can be attributed to their three-dimensional hierarchical structure. As we know, the morphology can affect the photocatalytic activity of photocatalysts. Three-dimensional hierarchical structures are regarded to have a higher superficial area and a greater number of active sites than either one-dimensional or two-dimensional architectures. Furthermore, for the three-dimensional flower-like octagonal crystals as shown in Figure 3b,c, all the surfaces of the steps on the petals are [100], [010], or [001] direction facets. And it has been demonstrated that the [100] facets are more reactive toward dissociative adsorption of reactant molecules compared with [101] facets, and crystals of exposed [001] facets exhibit much higher photocatalytic activity than the exposed [101] [13-17]. In addition, for flower-like AgCl samples, the faces mainly exposed on the petals are the [100] crystal facet system. Therefore, high photocatalytic efficiency is achieved for the flower-like AgCl microstructure with [100] facets.

## Conclusions

In summary, flower-like octagonal AgCl microstructures with enhanced photocatalysis are synthesized by a facile one-pot hydrothermal process for the first time. We investigate the evolution process of flower-like AgCl microstructures, including dendritic crystals’ fragmentizing, assembling, dissolving, and recrystallizing. Furthermore, flower-like AgCl microstructures exhibit enhanced photocatalytic degradation of methyl orange under sunshine. It is believed that the flower-like AgCl microstructures has potential application in the degradation of organic contaminations and disinfection of water, as well as in photovoltaic cells and other optoelectronic devices.

## Competing interests

The authors declare that they have no competing interests.

## Authors’ contributions

ML carried out the mechanism analysis and drafted the manuscript. HY investigated the preparation and characterization of the novel structures, and drafted the manuscript. RH carried out parts of the materials preparations. FB, MT, DS, BJ, and YL participated in the sequence analysis and discussion of the work. All authors read and approved the final manuscript.

## References

[B1] Wang P Huang BB Lou ZZ Zhang XY Qin XY Dai Y Zheng ZK Wang XN Synthesis of highly efficient Ag@AgCl plasmonic photocatalysts with various structures Chem Eur J 2010 8 538 544 10.1002/chem.200901954 19918815

[B2] Lou ZZ Huang BB Qin XY Zhang XY Cheng HF Liu YY Wang SY Wang JP Dai Y One-step synthesis of AgCl concave cubes by preferential overgrowth along <111> and <110> directions Chem Commun 2012 8 3488 3490 10.1039/c2cc30766a 22388463

[B3] Xu H Li HM Xia JX Yin S Luo ZJ Liu L Xu L One-pot synthesis of visible-light-driven plasmonic photocatalyst Ag/AgCl in ionic liquid ACS Appl Mater Interfaces 2011 8 22 29 10.1021/am100781n 21189040

[B4] An CH Peng S Sun YG Facile synthesis of sunlight-driven AgCl: ag plasmonic nanophotocatalyst Adv Mater 2010 8 2570 2574 10.1002/adma.200904116 20455207

[B5] Wang P Huang BB Qin XY Zhang XY Dai Y Wei JY Whangbo MH Ag@AgCl: a highly efficient and stable photocatalyst active under visible light Angew Chem Int Ed 2008 8 7931 7933 10.1002/anie.200802483 18773395

[B6] Kim S Chung H Kwon JH Yoon HG Kim W Facile synthesis of silver chloride nanocubes and their derivatives Bull Korean Chem Soc 2010 8 2918 2922 10.5012/bkcs.2010.31.10.2918

[B7] Han L Wang P Zhu CZ Zhai YM Dong SJ Facile solvothermal synthesis of cube-like Ag@AgCl: a highly efficient visible light photocatalyst Nanoscale 2011 8 2931 2935 10.1039/c1nr10247h 21611675

[B8] Lou ZZ Huang BB Wang P Wang ZY Qin XY Zhang XY Cheng HF Zheng ZK Dai Y The synthesis of the near-spherical AgCl crystal for visible light photocatalytic applications Dalton Trans 2011 8 4104 4110 10.1039/c0dt01795g 21384009

[B9] Ma YR Qi LM Ma JM Cheng HM Hierarchical, star-shaped PbS crystals formed by a simple solution route Cryst Growth Des 2004 8 351 354 10.1021/cg034174e

[B10] Fang JX Hahn H Krupke R Schramm F Scherer T Ding BJ Song XP Silver nanowires growth via branch fragmentation of electrochemically grown silver dendrites Chem Commun 2009 1130 1132 10.1039/b819003h 19225659

[B11] Zhang Q Liu SJ Yu SHJ Recent advances in oriented attachment growth and synthesis of functional materials: concept, evidence, mechanism, and future Mater Chem 2009 8 191 207 10.1039/b807760f

[B12] Kuai L Geng BY Chen XT Zhao YY Luo YC Facile subsequently light-induced route to highly efficient and stable sunlight-driven Ag-AgBr plasmonic photocatalyst Langmuir 2010 8 18723 18727 10.1021/la104022g 21114257

[B13] Selloni A Anatase shows its reactive side Nat Mater 2008 8 613 615 10.1038/nmat2241 18654584

[B14] Vittadini A Selloni A Rotzinger FP Gratzel M Structure and energetics of water adsorbed at TiO2 anatase s101d and s001d surfaces Phys Rev Lett 1998 8 2954 2957 10.1103/PhysRevLett.81.2954

[B15] Zheng ZK Huang BB Wang ZY Guo M Qin XY Zhang XY Wang P Dai YJ Highly efficient photocatalyst: tiO2 microspheres produced from TiO2 nanosheets with a high percentage of reactive {001} facets Phys Chem C 2009 8 14448 14453 10.1021/jp904198d 19876983

[B16] Tilocca A Selloni AJ Methanol adsorption and reactivity on clean and hydroxylated anatase (101) surfaces Phys Chem B 2004 8 19314 19319 10.1021/jp046440k

[B17] Yang HG Sun CH Qiao SZ Zou J Liu G Smith SC Cheng HM Lu GQ Anatase TiO2 single crystals with a large percentage of reactive facets Nature 2008 8 638 642 10.1038/nature06964 18509440

